# Resource heterogeneity and foraging behaviour of cattle across spatial scales

**DOI:** 10.1186/1472-6785-9-9

**Published:** 2009-04-24

**Authors:** Santiago A Utsumi, Carlos A Cangiano, Julio R Galli, Mary B McEachern, Montague W Demment, Emilio A Laca

**Affiliations:** 1 Department of Animal and Rangeland Sciences, New Mexico State University, Las Cruces, NM 88003, USA; 2 Estación Experimental Balcarce, Instituto Nacional de Tecnología Agropecuaria, 7620 Balcarce, Buenos Aires, Argentina; 3 Facultad de Ciencias Agrarias, Universidad Nacional de Rosario, 2123 Zavalla, Santa Fe, Argentina; 4 Department of Plant Sciences, University of California, Davis, CA 95616, USA

## Abstract

**Background:**

Understanding the mechanisms that influence grazing selectivity in patchy environments is vital to promote sustainable production and conservation of cultivated and natural grasslands. To better understand how patch size and spatial dynamics influence selectivity in cattle, we examined grazing selectivity under 9 different treatments by offering alfalfa and fescue in patches of 3 sizes spaced with 1, 4, and 8 m between patches along an alley. We hypothesized that (1) selectivity is driven by preference for the forage species that maximizes forage intake over feeding scales ranging from single bites to patches along grazing paths, (2) that increasing patch size enhances selectivity for the preferred species, and that (3) increasing distances between patches restricts selectivity because of the aggregation of scale-specific behaviours across foraging scales.

**Results:**

Cows preferred and selected alfalfa, the species that yielded greater short-term intake rates (P < 0.0001) and greater daily intake potential. Selectivity was not affected by patch arrangement, but it was scale dependent. Selectivity tended to emerge at the scale of feeding stations and became strongly significant at the bite scale, because of differences in bite mass between plant species. Greater distance between patches resulted in longer patch residence time and faster speed of travel but lower overall intake rate, consistent with maximization of intake rate. Larger patches resulted in greater residence time and higher intake rate.

**Conclusion:**

We conclude that patch size and spacing affect components of intake rate and, to a lesser extent, the selectivity of livestock at lower hierarchies of the grazing process, particularly by enticing livestock to make more even use of the available species as patches are spaced further apart. Thus, modifications in the spatial pattern of plant patches along with reductions in the temporal and spatial allocation of grazing may offer opportunities to improve uniformity of grazing by livestock and help sustain biodiversity and stability of plant communities.

## Background

Spatial patchiness of forage often results in uneven livestock grazing, with potential implications for patterns of forage intake, resource degradation, and plant community composition and invasibility [1]. Grazing concentrated in patches of preferred forage can lead to patch degradation over time. Improved grazing uniformity and the prevention of heavy, uneven grazing in patches of preferred forage is a primary goal of sound grazing management [2]. However, a better understanding of the mechanisms governing forage preference and selectivity in patchy, heterogeneous environments is needed to inform new management approaches that enhance grazing distribution at spatial sales ranging from bites to patches [3].

Preference and selectivity are related but different terms that describe patterns of forage selection. Preferences are traditionally determined by offering equally accessible amounts of alternative forage and comparing forage intake. Thus, preference is a term that describes the individual's forage intake in the absence of any constraints on availability or accessibility [4]. Selectivity, on the other hand, is a measure of forage intake under patchy, heterogeneous conditions where alternative forages are not equally available or uniformly distributed [5]. In this context, selection of preferred forage can often be constrained by environmental and physiological trade-offs [4,5]. Thus, depending on the trade-offs involved, selectivity will not necessarily coincide with preference.

Grazing ungulates exhibit complex patterns of selectivity in heterogeneous environments with several forage species [6]. Selectivity patterns often agree with the intake rate-maximization hypothesis [7,8], which predicts both a matching of patch selection and intake rate [9-14], and behavioral strategies to overcome the constraints impose by spatial heterogeneity on preferences [15-17]. According to this hypothesis, energy, protein, minerals and food mass are some of the foraging currencies. Patch residence time, travel speed, and search patterns are some of the decision variables and mechanisms by which animals achieve a certain foraging success [18,19]. Foraging theory predicts that selectivity increases with increasing difference in profitability (e.g. nutrient intake) among options. When patch encounter rate declines, selectivity is also expected to decline. [8].

Theoretical models [3,20,21] suggest emerging selectivity patterns from behaviors that operate across several nested foraging scales. Selectivity can occur at the scale of a bite, feeding station, patch, or feeding site [3] depending on how behaviors with regard to bite formation (tongue, lips and jaw movements) and movements between successive bite locations (head and neck movements), feeding stations (single step), and patches (several steps) take place. This nested nature of foraging suggests that selectivity could result from differential behaviors at separate feeding scales and may accumulate with the continuation of foraging events across feeding scales [22].

Spatial heterogeneity in the quantity and quality of food and forage depletion are major determinants of foraging behaviors across these scales [20,21]. Structural characteristics of single plants within a patch can affect the functional response of instantaneous intake rate through effects on bite mass and handling time [23]. Increased rates of forage depletion will tend to decrease residence times in patches [24]. Patch size relative to animals [25,22] and distances between patches [26] can also affect grazing patterns within and between patches through effects on patch selection and through rewards and costs of grazing at the current patch [1]. Thus, patchiness across a foraging landscape can force herbivores to trade-off preference and diminishing rewards at a current patch undergoing depletion against the travel cost of searching for a new undepleted patch [27,28]. Selectivity of preferred forage should decrease when rewards relative to travel distance decrease as patches are spaced further apart [28]. Average intake rates should decline with increasing distance between patches, whereas time spent foraging and the number of bites per patch should correlate positively with the size of patches and the distance between them [29,30]. If patch size and distance are important determinants of foraging patterns, changes in their size and spacing should have predictable effects on selectivity of grazing livestock.

The goal of this study was to quantify the effects of spatial patchiness and scale of foraging on ingestive behavior and selectivity of cattle. Following Charnov's [27] marginal value theorem, we hypothesized that cows adopt a foraging strategy that maximizes intake rate, however, selectivity for preferred species will be constrained by the spatial heterogeneity of forage. The following associated predictions were tested: 1) grazers prefer the forage species that yields higher intake rates, 2) selectivity for preferred species increases with increasing patch size but decreases with increasing distance between patches, 3) selectivity for preferred species increases as more feeding events are integrated across feeding scales, 4) average dry matter intake rate decreases as distance between patches increases, and 5) patch residence time increases with patch size and as distance between patches increases.

## Results

Tall fescue plants were shorter and had more mass per pot than alfalfa plants (Table 1). Tall fescue herbage had lower IVDMD%, higher CP%, higher DM% and higher NDF% than alfalfa (Table 1). Both species sustained similar biting rates, but alfalfa plants yielded larger bites and greater intake rates (Table 1). Based on differences in NDF%, it was estimated that alfalfa plants provided a potential digestible daily intake 56% higher than fescue (Table 1).

**Table 1:** Characteristics of tall fescue and alfalfa plants (± SE) with significance (P < 0.05) for difference between species (*NDF*, neutral detergent fiber; *DMIVD*, dry matter *in vitro *digestibility; *IR*, dry matter intake rate; *DI*, digestible dry matter intake).

	Forage species		*P *value
		
	Alfalfa	Tall fescue	
Mass offered (g/pot)	3.69 (± 0.25)	4.66 (± 0.25)	< 0.0093
Height (cm)	29.9 (± 0.2)	19.8 (± 0.2)	< 0.0001
Dry matter (%)	22.8 (± 0.6)	25.1 (± 0.6)	< 0.0073
Crude protein (%)	22.7 (± 0.5)	12.5 (± 0.5)	< 0.0001
NDF (%)	33.2 (± 0.9)	51.9 (± 0.9)	< 0.0001
DMIVD (%)	71.7 (± 0.9)	66.5 (± 0.9)	< 0.0001
Average bite weight (g)	1.55 (± 0.03)	0.89 (± 0.03)	< 0.0001
Average biting rate (bites/min)	45.0 (± 1.0)	46.2 (± 1.0)	NS^1^
Average IR (g/min)	69.7 (± 1.6)	41.07 (± 1.6)	< 0.0001
Maximum daily DI (kg)	20.55	13.12	Not determined

^1^NS: Not significant.

Cows showed a significant preference (1.74 ± 0.09) and selectivity (1.47 ± 0.04) for alfalfa. As expected, preferences were constrained under the 9 treatments, resulting in a lower selectivity index compared to preference (Table 2). Patch size and distance between patches did not affect either partial or cumulative selectivity. When examining selectivity along separate steps of the grazing process, significant partial selectivity occurred, but only at the bite scale due to differences in bite mass between plant species. Partial selectivity was also observed at the feeding station scale (Table 2). When integrating behaviors along the grazing process, cumulative selectivity increased from patch encounters to total intake, but became significant only when bite formation and mass were included as final steps in the grazing process (Table 2).

**Table 2:** Indices (± s.e) of partial and cumulative selectivity for alfalfa at each separate scale and across scales of the grazing process with the correspondent significance for selectivity and for the effect of patch size, distance between patches, and interaction between patch sizes and distances.

		Significance (p value)			
		
	Mean (± s.e)	Selectivity	Patch size	Distance	Patch size × Distance
**Partial Selectivity**					
Patch selection	1.01 (± 0.02)^b^	0.44	0.47	0.44	0.56
Feeding stations per patch	1.04 (± 0.02) ^b^	0.07	0.84	0.68	0.90
Bites per feeding station	0.99 (± 0.02) ^b^	0.73	0.85	0.38	0.82
Dry matter intake per bite	1.26 (± 0.02) ^a^	< 0.0001	0.68	0.41	0.29

**Cumulative Selectivity**					
Patches grazed (%)	50.7 (± 1.45) ^b^	0.60	0.47	0.44	0.56
Feeding stations visited (%)	52.4 (± 1.45) ^b^	0.09	0.43	0.24	0.43
Bites cropped (%)	52.3 (± 1.45) ^b^	0.12	0.64	0.16	0.52
Total dry matter intake (%)	65.2 (± 1.45) ^a^	< 0.0001	0.76	0.27	0.67

Partial or cumulative selectivity indexes with different superscript are significantly different according to Duncan test. N = 25.

**Figure 1:**
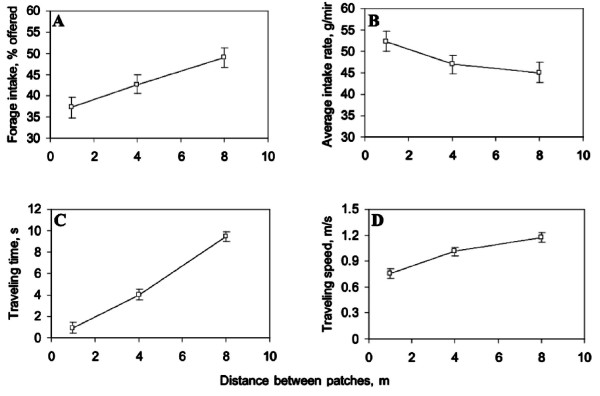
**Behavioral response of cattle to the distance between patches: A) Forage intake expressed as the percent of forage offered, B) average dry matter intake rate, C) travel time, and D) travel speed as a function of the distance between patches**. Values are least square means of 3 cows. Vertical bars denote standard errors.

Average intake rate declined linearly whereas travel time, speed, and proportion of total herbage consumed increased linearly with linear increases in the distance between patches (Fig. 1). No patch size effects or interactions between patch size and distance were observed.

At the patch level, cows had similar behavioral responses to patch size and distance between patches in both alfalfa and fescue patches (Table 3 and 4). No interaction between factors was detected. We observed positive linear effects of patch size on dry matter intake, number of bites, and residence time per patch for both species. As patch size increased, cows increased the number of feeding stations visited per patch, but not the number of bites taken per feeding station (Tables 3 and 4). Roughly 2, 4, and 6 feeding stations per patch were observed for small, medium, and large patches, respectively (Table 4). The number of bites per feeding station ranged from 5–6 regardless of patch size or species (Table 4). When cows foraged on small and large patches of alfalfa, they cropped heavier bites and achieved higher intake rates compared to medium patches. When cows foraged on fescue patches, bite weight and intake rate were not affected by patch size.

**Table 3:** Summary of probability values for the effect of patch size and distance between patches and for the linear and quadratic effect on components of feeding behavior by cows at alfalfa and tall fescue patches.

	Patch size			Distance between patches		
		
	ANOVA	Linear	Quadratic	ANOVA	Linear	Quadratic
Dry matter intake (g)						
Alfalfa	< 0.0001	< 0.0001	0.164	0.117	0.065	0.349
Fescue	< 0.0001	< 0.0001	0.493	0.087	0.031	0.901
Bites cropped						
Alfalfa	< 0.0001	< 0.0001	0.429	0.037	0.011	0.785
Fescue	< 0.0001	< 0.0001	0.159	0.021	0.008	0.566
Residence time (s)						
Alfalfa	< 0.0001	< 0.0001	0.161	0.047	0.015	0.996
Fescue	< 0.0001	< 0.0001	0.288	0.047	0.026	0.328
Feeding stations (FS)						
Alfalfa	< 0.0001	< 0.0001	0.154	0.516	0.363	0.543
Fescue	< 0.0001	< 0.0001	0.286	0.053	0.042	0.279
Bites per FS						
Alfalfa	0.627	0.305	0.714	0.342	0.142	0.867
Fescue	0.196	0.245	0.156	0.012	0.004	0.587
Bite weight (g)						
Alfalfa	0.010	0.223	0.004	0.151	0.155	0.212
Fescue	0.175	0.197	0.161	0.409	0.318	0.426
Biting rate (bites/min)						
Alfalfa	0.073	0.025	0.857	0.692	0.408	0.928
Fescue	0.771	0.983	0.478	0.374	0.168	0.905
Intake rate (g/min)						
Alfalfa	0.040	0.478	0.017	0.505	0.487	0.377
Fescue	0.106	0.067	0.233	0.528	0.666	0.313

There were no significant interactions between patch sizes and distances between patches.

**Table 4:** Ingestive behavior of cows within alfalfa and tall fescue patches of three different size spaced with three different distances between patches. between patches.

	Patch size			Distance between patches		
		
	Small	Medium	Large	1 m	4 m	8 m
Dry matter intake (g)						
Alfalfa	18.3 ± 2.4^c^	35.9 ± 2.3^b^	58.47 ± 2.2^a^	35.8 ± 2.3	36.5 ± 2.2	40.4 ± 2.4
Fescue	10.2 ± 4.7^c^	23.0 ± 4.5^b^	32.5 ± 4.3^a^	17.9 ± 4.4	21.8 ± 4.3	26.0 ± 4.6
Bites cropped						
Alfalfa	11.1 ± 1.2^c^	25.6 ± 1.2^b^	37.1 ± 1.1^a^	22.1 ± 1.2^b^	24.5 ± 1.1^ab^	27.2 ± 1.2^a^
Fescue	10.7 ± 3.8^c^	27.6 ± 3.6^b^	36.6 ± 3.4^a^	19.5 ± 3.5^b^	25.8 ± 3.4^ab^	29.6 ± 3.7^a^
Residence time (s)						
Alfalfa	15.3 ± 1.5^c^	34.2 ± 1.4^b^	46.9 ± 1.3^a^	28.8 ± 1.4^b^	32.4 ± 1.3^ab^	35.3 ± 1.5^a^
Fescue	13.9 ± 4.2^c^	34.6 ± 4.0^b^	47.8 ± 3.9^a^	25.5 ± 3.9^b^	34.2 ± 3.8^ab^	36.6 ± 4.2^a^
Feeding stations (FS)						
Alfalfa	2.1 ± 0.2^c^	4.5 ± 0.2^b^	6.5 ± 0.2^a^	4.3 ± 0.2	4.5 ± 0.2	4.4 ± 0.2
Fescue	2.0 ± 0.2^c^	4.3 ± 0.2^b^	6.3 ± 0.2^a^	4.0 ± 0.2^b^	4.3 ± 0.2^ab^	4.6 ± 0.2^a^
Bites per FS						
Alfalfa	5.1 ± 0.4	5.7 ± 0.4	5.7 ± 0.4	5.0 ± 0.4	5.5 ± 0.4	6.0 ± 0.4
Fescue	5.2 ± 0.5	6.2 ± 0.5	5.8 ± 0.5	4.6 ± 0.5^b^	5.8 ± 0.4^ab^	6.7 ± 0.5^a^
Bite weight (g)						
Alfalfa	1.65 ± 0.12^a^	1.42 ± 0.12^b^	1.57 ± 0.12^ab^	1.61 ± 0.12	1.50 ± 0.12	1.53 ± 0.12
Fescue	0.97 ± 0.10	0.82 ± 0.10	0.87 ± 0.10	0.95 ± 0.10	0.85 ± 0.10	0.86 ± 0.10
Biting rate (bites/min)						
Alfalfa	43.3 ± 1.5^b^	44.8 ± 1.3^ab^	47.5 ± 1.2^a^	44.7 ± 1.3	45.2 ± 1.2	45.7 ± 1.5
Fescue	47.2 ± 2.1	47.6 ± 1.9	46.1 ± 1.7	45.8 ± 1.9	46.2 ± 1.7	48.9 ± 2.1
Intake Rate (g/min)						
Alfalfa	71.5 ± 5.7^ab^	63.7 ± 5.6^b^	74.4 ± 5.5^a^	72.3 ± 5.5	67.9 ± 5.4	69.4 ± 5.7
Fescue	46.2 ± 5.7	39.3 ± 5.7	39.6 ± 5.6	43.4 ± 5.7	39.8 ± 5.6	41.9 ± 5.7

Main effect least square means with different superscript are significantly different according to LSD _0.05_. There were no significant interactions between patch sizes and distances

The number of bites and residence times in alfalfa and fescue patches increased linearly as distance between patches increased. A similar but weaker trend was detected in dry matter intake (Table 3). With an increase in distance from 1–8 m, bites and residence time increased by 23% in alfalfa patches, while bites, residence time, and intake increased by 52%, 44%, and 45%, respectively, in fescue patches (Table 4). As distance increased, cows tended to visit more feeding stations per patch and take more bites per feeding station in fescue, but not alfalfa, patches (Tables 3 and 4). Though not statistically significant, bite weight and intake rate decreased 4–5% in alfalfa, and 3–9% in fescue patches as distances increased from 1–8 m (Table 4). Biting rates within alfalfa and fescue patches were not significantly affected by distance between patches, but biting rate in alfalfa tended to increase linearly as patch size increased (Table 3 and 4).

## Discussion

### Foraging behavior and intake maximization

In agreement with expectations, cows exhibited preference and selectivity for alfalfa, the species associated with higher forage quality, heavier bite weights and greater intake rate and potential daily forage intake (Prediction 1). However, contrary to Prediction 2, selectivity was not significantly influenced by differences in patch size or distance. As expected, selectivity was dependent on scale-specific behaviors and tended to increase with the integration of behaviors across foraging scales (Prediction 3). Average dry matter intake rate decreased as distance between patches increased (Prediction 4), whereas patch residence times increased with increasing size and distance between patches (Prediction 5). Overall, our findings support the conclusion that when confronted with heterogeneous, patchy environments of contrasting plant species, cows grazed in a manner consistent with the "intake rate-maximization" hypothesis in two ways. First, over the integration of foraging scales, cows selected the preferred species that yielded higher intake rates. Second, in response to travel time constraints, cows were able to modify behaviors within and between patches to compensate for decreases in average intake rate.

We attempted to expose cows to a clear trade-off between quantity and quality whereby more restrictive foraging conditions (greater distance and smaller patches) would motivate lower selectivity for the preferred forage in order to achieve maximum daily intake or instantaneous intake rate [31]. The trade-off obtained did not affect selectivity, with the exception that preference for alfalfa was much greater than selectivity. However, the fact that selectivity in all of the patchy environments was much lower than the preference for alfalfa indicates that greater restrictions to intake can reduce selectivity. Contrary to studies where spatial scale of patchiness readily affected selectivity patterns of herbivores [15,22,25], lack of response in this study may be influenced by our experimental design. Forcing cows to graze in a linear path along an alley where encounters with plant species are strictly sequential may limit the ability of cows to orient their grazing paths toward preferred patches.

In this study, cows exhibited three mechanisms that adjust intake rate and foraging efficiency in response to changes in a patchy food environment. First, cows selected more alfalfa over fescue when patches were arranged side-by-side (preference tests) than when arranged sequentially in monospecific patches (selectivity tests). Second, patch residence time and depletion [24] increased with increasing distance between patches [26]. Third, travel speed between patches also increased with increasing distance between patches, a compensatory response related to the period of acceleration and deceleration associated with each patch [32].

### Patch depletion and residence time

The greater increase in patch residence times with increasing distance between patches in fescue compared to alfalfa patches reflects the predominant role of sward structure and is also consistent with a strategy linearly related to maximization of intake rate. Tall sparse swards exhibit faster depression of within-patch intake rate with increasing patch depletion than short dense ones, with important implications for patch time residence [24,30,33]. Foragers maximizing intake rate should respond to an increase of travel time between patches by increasing residence time more in patches with slow (e.g. fescue) than in patches with rapid depression (e.g. alfalfa).

As patch size increased (4, 8 and 12 pots), the number of feeding stations per patch increased in direct proportion (2, 4, and 6 feeding stations per patch) whereas bites per feeding station and bite weight were not significantly different, with the exception that bite weight was smaller in medium than in small alfalfa patches. This suggests that patches were essentially depleted in a systematic manner by horizons and provides an experimental basis for scaling up patch depression curves in models of grazing behavior in heterogeneous environments with variable patch sizes [34].

### Foraging scales

Selectivity for alfalfa was a scale-dependent process that emphasized the bite formation level. Grazing is a nested process where forage selection emerges from various behaviors at several feeding scales [3]. Selectivity can increase as temporal and spatial scales expand [22]. In this study, cows exhibited partial selectivity for alfalfa by visiting more feeding stations per patch of alfalfa compared to fescue, but mostly by obtaining larger bites from alfalfa (Table 2). Whether or not these components of selectivity are best interpreted as a result of structural constraints of plants on bite formation or as true changes in feeding motivation is debatable. Bite weight seems to be more sensitive to the structural effects of local sward attributes, while behaviors at higher levels in the hierarchy (i.e. patches, feeding sites, home ranges) are more dependent on changes in feeding motivation driven by integrated responses to large scale biotic and abiotic heterogeneity [1,3,35]. This nested nature of grazing suggests the need to further refine quantitative approaches to better understand the influence of plant structural and animal volitional factors likely to result in patterns of grazing selectivity. This study suggests that at least within the range of bites to patches, selective behaviors are likely to accumulate across scales.

## Conclusion

Manipulation of foraging selectivity of livestock is a key to managing their impact on the landscape, but it is difficult. Whether in pastures or rangelands, major problems often result from uneven grazing and its effects on plant demography, competition and community structure. Traditional grazing management schemes are not based on a mechanistic understanding of the grazing process, and therefore, have not evolved to keep up with the changing needs of ecosystem management.

Selectivity can increase as more behavioral steps and spatial levels are involved. Therefore, total selectivity and relative impact on a preferred patch type or plant species can be reduced by limiting the numbers of levels available for selection. This can be achieved by reducing the available area and or time for grazing. The effect of reduced levels of selectivity can complement the traditional method to obtain even grazing of all patches by forcing animals to deplete available forage.

Differential defoliation of plant species is strongly linked to the effects of plant architecture on bite weight. These effects seem to be "hard wired" and show little susceptibility to manipulation of foraging behavior. Other things being equal, taller plants yield larger bites and are defoliated more severely. This work provides a better understanding and quantification of components and mechanisms of cattle selectivity in heterogeneous environments that can support the development of novel grazing management methods.

## Methods

Experiments were conducted at the Experimental Field J.F. Villarino, Facultad de Ciencias Agrarias, Universidad Nacional de Rosario, Argentina (33.01° L.S.; 60.53° L.W.) during February and March of 2001. We used 3 non-lactating pregnant Holstein dairy cows (620 ± 81 kg) with experience grazing on patches created by groups of 6-liter pots with either alfalfa or fescue. Cows were maintained on mixed paddocks of alfalfa and tall fescue near the experimental site and were subjected to a 1-hour fast before measurements.

We used 30-cm tall alfalfa and 20-cm tall fescue plants for all grazing experiments. Alfalfa was in early flower, while fescue plants were in vegetative stage. Herbage mass per pot was determined by clipping 3 pots of each species in each grazing session at 2 cm above the pseudostems in tall fescue and above the crown in alfalfa. Herbage samples were dried at 60°C to determine dry matter content (DM%) and analyzed for neutral detergent fiber (NDF%) [36], crude protein (CP%) [37] and in vitro dry matter digestibility (IVDMD%) [38].

To determine preferences, cows were exposed to an experimental grazing area (8 × 2 m) containing 16 pots of each species on each side of a 0.65 m corridor. Pairs of pots were fixed to the ground with double iron holders and were identified with metallic tags to facilitate data recording. Preference sessions consisted of allowing each cow to graze 35 bites in isolation. This process was repeated 4 times with each cow, twice with alfalfa on the left and twice with alfalfa on the right, generating a total of 12 preference sessions. The order of sessions was determined at random and each lasted 1–2 minutes. All preference sessions were carried out in the first two days of the study.

**Figure 2:**
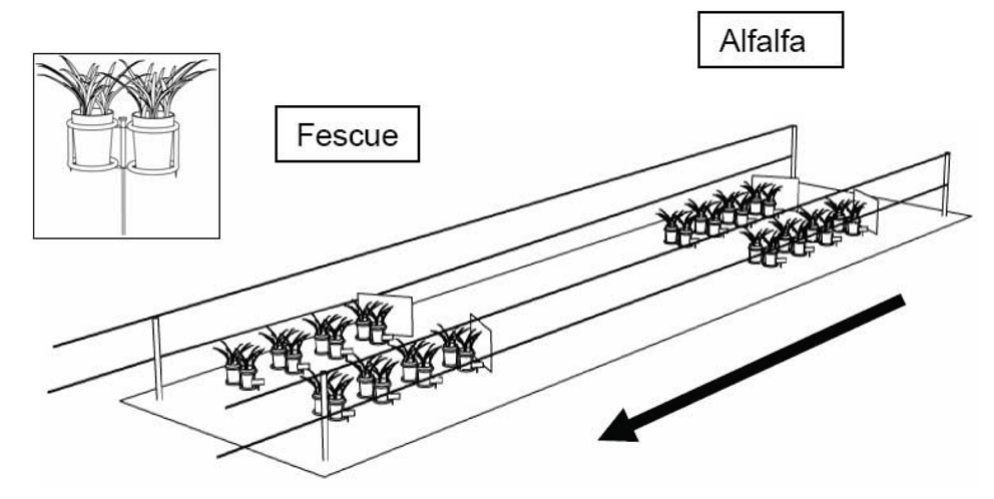
**Segment of a foraging environment created by alternating patches of alfalfa and fescue along an alley (100 m long × 2 m wide) used to test the selectivity of cows in response to size of and distance between patches**. The arrow indicates the direction of grazing.

Following preference sessions, we examined selectivity by exposing each cow to experimental treatments with different patch sizes and distributions of species. We created 9 treatments using a factorial combination of 3 patch sizes (small, medium and large) and 3 distances between patches (1, 4 and 8 m). We used 8, 16, and 24 pots to create each small, medium, and large patch, respectively. Patches were created by arranging pots in pairs along the sides of the alley with a central corridor (0.65 m) remaining for the transit of cows (Fig. 2). Patches were arranged in a linear sequence along a fenced alley of 100 × 2 m such that successive patches were alternating sets of alfalfa and tall fescue patches. Opaque plastic panels (0.45 × 0.50 m) were placed in front of patches to prevent cows from seeing available patches from a distance. Treatments, therefore, varied in number of pots and patches, length of corridor, area covered by patches, total herbage mass, and mass of herbage offered per unit area (Table 5).

**Table 5:** Main characteristics of scenarios with patches of plant pots of alfalfa and fescue used to examine selectivity and feeding behavior by cattle.

	Small			Medium			Large		
			
	1 m	4 m	8 m	1 m	4 m	8 m	1 m	4 m	8 m
Total number of pots^1^	144	144	96	128	128	128	144	144	144
Patches^1^	18	18	12	8	8	8	6	6	6
Distance covered (m)	26	77	94	19	40	68	20	35	55
Area covered (m^2^)	52	136	188	38	80	136	40	70	110
Total mass offered (g)	631 ± 66	593 ± 66	408 ± 54	527 ± 59	527 ± 59	544 ± 72	631 ± 66	631 ± 66	631 ± 66
Mass per area (g/m^2^)	12 ± 1.3	4 ± 0.4	2 ± 0.3	14 ± 1.5	7 ± 0.7	4 ± 0.5	16 ± 1.7	9 ± 1.0	6 ± 0.6

^1^Half of the pots and half of the patches had only alfalfa and the other half contained only fescue.

Scenarios were the factorial combination of three patch sizes and three distances between patches. Mean value for total number of pots and patches, distance and area covered over the alley, and total mass and mass offered per unit area are presented. Standard deviation of means is also given for total mass and mass offered per unit area.

During each selectivity session a single cow entered one end of the experimental area and grazing until she reached the other end. We conducted an average of three selectivity sessions per day, the first starting near sunrise and the last finishing near sunset. Because the number of pots was limited, we completed this experiment in two periods, utilizing plant re-growth in the second period. Each cow grazed each treatment only once. A total of 27 selectivity sessions were conducted and each one lasted 3–7 minutes.

Foraging behavior was recorded with a camcorder (Sony^® ^Handycam 20 ×) and data were used to characterize selectivity across 4 nested foraging scales: bite, feeding station, patch and treatment. Similar to Bailey et al. [3] the bite scale was defined by the sequence of herbage prehension, jaw and tongue movements, and severance of forage by head movement. The feeding station was defined by the arrangement of plant pots immediately available to a cow from were one or more bites were cropped without moving its front feet. The patch scale was defined by the spatial arrangement of neighboring plant pots of the same plant species. Finally, the treatment was defined by the sequential arrangement of patches over the experimental alley. We measured the number of bites, number of feeding stations, number of patches grazed, time spent foraging, and speed of movement in each treatment session. Time spent foraging was partitioned into patch residence time and travel time between patches. Residence time per patch was total residence time divided by number of patches grazed. Speed of movement was the quotient of the distance traveled and time spent traveling between patches.

We calculated dry matter intake, intake rate, biting rate, and bite weight for each plant species and patch. Potential maximum digestible daily intakes of each species were also estimated using a fill constrain of 11 g NDF/kg of body weight [39]. Dry matter intake was estimated by the difference between initial and final weights of the grazed pots, corrected for water loss, and multiplied by dry matter content of the forage. Water loss by pots was determined for each grazing session by linear regressions of pot weight (y) against time of weighing (x) of 5 intact and 5 defoliated pots randomly selected and weighed at intervals of 5 to10 minutes. Dry matter intake at the scenario level was expressed as proportion of mass removed due to differences in the mass offered by treatments (Table 5).

Bite weight (g) was dry matter intake divided by number of bites cropped of each species. Biting rate (bites/min) was the number of bites cropped divided by residence time. Intake rate within patches (g/min) was the quotient of dry matter intake and residence time. Average intake rate at the scenario level (g/min) was the quotient of total dry matter intake and total time spent foraging.

### Data Analysis

Preference and selectivity indices were calculated as ratios of the proportion of alfalfa in the diet and the proportion of alfalfa in the dry matter offered. Significant preference and selectivity for alfalfa were indicated by values greater than 1 (student's t-test, ∝ = 0.05). Similar to WallisDe Vries et al. [22], selectivity was also analyzed with a hierarchical nested approach at each and across the 4 foraging scales of interest. We calculated indices of partial and cumulative selectivity for alfalfa based on behaviors particular to each separate feeding scale and for the integration of ongoing behaviors across the feeding scales (Table 6). Significant partial and cumulative selectivity for alfalfa were indicated by values greater than 1 (Student's t-test, α = 0.05).

**Table 6:** Foraging events and equations used in the calculation of partial and cumulative indexes of selectivity for alfalfa at each separate scale and across scales of the grazing process.

Foraging scale	Foraging event	Partial index	Cumulative index
Scenario	Patches selected (Ps)		
Patch	Feeding stations visited (FS)		
Feeding station	Bites cropped (B)		
Bite	Dry matter intake (I)		

Partial selectivity indexes expressed the ratio of the proportion of foraging events on alfalfa at a focal scale and those at the next larger scale of the grazing process. Cumulative selectivity indexes showed a progressive integration of the proportion of foraging events on alfalfa against the proportion of alfalfa patches offered.

Preference and selectivity data were analyzed using a mixed linear model with the MIXED procedure [40] and the Kenward-Roger method for degrees of freedom. Preference was analyzed with the following model:



Where Y_ijk _is the preference value observed, μ is the general mean, α_i _is the random effect of cows, β_j _is the fixed effect of side on which alfalfa was offered, and ∈_ijk _is the residual.

Selectivity data were analyzed with the following model:



Where Y_ijklm _is the observed selectivity, μ is the general mean, α_i _is the random effect of cows, β_j _is the random effect of periods, γ_k _is the fixed effect of patch size, δ_l _is the fixed effect of distance between patches, (γ * δ)_kl _is the fixed effect of the interaction between patch size and distances, and ∈_ijklm _is the residual. Pre-established contrasts for linear and quadratic effects of patch size and distances were also assessed. Differences on least square means were performed using the LSD test. Student's t-test was used to compare preference and selectivity for alfalfa exhibited by cows. This comparison used single preference and selectivity sessions as independent replicates.

## Authors' contributions

SAU conducted most of the field work, data collection and initial analysis, and wrote the first draft. CAC helped design and implement the experiment, and wrote the second version of the manuscript. JRG provided the logistic support, collected some data, and supervised field work. MBM edited the second, third, and forth versions of the manuscript and coordinated manuscript submission. MWD helped conceive the experiment, provided part of the financial support for the work, and provided editorial comments. EAL conceived the experiment and designed most of the setup, posed the hypotheses to be tested and wrote the final version of the manuscript after supervising the writing of the first and third versions. All of the authors read, provided additional editorial comments, and approved the manuscript.
